# Hepatitis B Virus Genotype G Infection in a Turkish Patient Undergoing Hemodialysis Therapy

**DOI:** 10.5812/hepatmon.826

**Published:** 2012-02-29

**Authors:** Murat Sayan, Cengiz Dogan

**Affiliations:** 1 University of Kocaeli, Faculty of Medicine, Clinical Laboratory, PCR Unit, Kocaeli, Turkey; 2 Fresenius Medical Care, Istanbul, Turkey

**Keywords:** Hepatitis B, Chronic, Hepatitis B virus, Genotype, DNA Sequence, Unstable, Genes, Pol

## Abstract

**Background:**

Genotype G is the least common of all the hepatitis B virus (HBV) genotypes. The existence of the genotype G strain of HBV was first noted in 2000 and little information is available on its global geographical distribution. Previous studies have demonstrated the dominance of genotype D in patients with HBV infections in Turkey.

**Objectives:**

To report for the first time in Turkey, the case of a 61 year old male patient who developed the HBV genotype G infection.

**Case report:**

According to HBV genotyping using phylogenetic analysis and an INNO-LiPA assay, the patient was infected with genotype G and G+A, respectively.

**Conclusions:**

The present clinical study suggests that the transmission of an HBV genotype other than genotype D, namely HBV genotype G, is possible in Turkey. Epidemiological and clinical information on genotype G infection is currently limited, and this is most likely due to its low prevalence throughout the world. Therefore, it may be important to determine the epidemiologic and molecular characteristics of the HBV genotype G as it relates to chronic hepatitis, to enable better understanding of its circulation and progression around the world.

## 1. Background

The existence of genotype G was first noted in 2000 and to date it has been localized in the United States, Canada, Brazil, Mexico, France, Germany, Vietnam, Thailand, and Japan [1][2][3][4]. Genotype G is essentially identical to the other HBV genotypes, but it has some unique features including a 36-bp insertion downstream of the core gene start codon. This results in a twelve amino acid (aa) insertion at the N-terminal end of the core protein, and two stop codons in the precore region that prevents the expression of HBeAg [1]. Genotype G is the most uncommon of all the HBV genotypes. This genotype is usually found as a coinfection with another HBV genotype [4]. However, epidemiological and clinical information on the genotype G infection is limited, which is probably the result of its low worldwide occurrence [3]. Previous studies have demonstrated the dominance of genotype D in patients chronically infected with HBV in Turkey [5][6]. Nevertheless, transmission of genotypes (genotype A2/adw2) other than genotype D HBV is also possible in Turkey [7]. In this study, we report on the case of a male patient who developed the HBV genotype G infection, and reveal its coinfection with other HBV genotypes for the first time in Turkey.

## 2. Case Report

The study patient was a 61 year old male diabetic who had been treated with hemodialysis three times per week for 4 years, due to idiopathic end stage renal disease. Twelve years earlier, the patient had been diagnosed with brucellosis. At that time, he was hospitalized and hepatitis B surface antigen (HBsAg) positivity was detected for the first time. No other members of his family were shown to have a hepatitis B infection on examination. The patient had never used intravenous drugs in the past and had no history of blood transfusion, nor had he ever traveled abroad. The patient did, however, have a history of multiple sexual partners. The laboratory analyses yielded; transaminase levels, ALT:41 U/L, AST:25 U/L; bilirubin levels, BU:0.1mg/dl, BC:0.3 mg/dl; HBsAg, positive (S/Co:3894); anti HBs antibody, negative; anti-HBcAg total antibody (anti-HBcIgG), positive; anti-HBcAg IgM antibody (anti-HBcIgM), negative; HBeAg antigen (HBeAg), negative; anti-HBe antibody (anti-HBe), positive; HBV DNA load, 5.94E+05 IU/ml; anti-HAV IgG, positive. Our case was characterized by seronegativity for HDV, HCV and HIV (he was also HIV-PCR negative) and with autoantibody negativity (anti-nuclear, anti-mitochondrial, anti-double stranded DNA and anti-smooth muscle antibodies). The patient did not give his consent for a liver biopsy procedure; therefore assessment of liver damage was not possible. The patient was evaluated as having "HBeAg - negative chronic hepatitis B," according to the European Association for the Study of the Liver (EASL)clinical practice guidelines, and he was treatment naive. Serological markers for HBV were tested using the commercially available microparticle enzyme immunoassay kit (AxSYM, Abbott Laboratories, IL, USA). HBV DNA was isolated from the serum sample on the bio-robot workstation, using magnetic-particle technology (QIAsymphony SP, QIAGEN GmbH, Hilden, Germany). HBV DNA was detected and quantified by a commercial PCR assay (artus HBV QS-RGQ test, QIAGEN GmbH, Hilden, Germany) on the real-time platform (Rotor-Gene Q, QIAGEN GmbH, Hilden, Germany). A pair of primers was designed (forward: 5'-TCGTG GTGGACTTCTCTCAATT-3' and reverse: 5'-CGTTGACAGACTTTCCAATCAAT-3') for amplification of the HBV pol gene region (pol gene sequence used routinely for HBV genotyping and genotypic resistance analysis to nucleos(t)ide analogues in our clinical laboratory). The PCR conditions were applied as previously described [7].

### 2.1. HBV Genotyping by DNA Sequencing

HBV genotype was determined by phylogenetic analysis. The nucleotide sequence was compared to those from the international DNA data bank (GenBank+EMBL+DDBJ+PDB). Phylogenetic comparison was performed by UPGMA analysis using the CLC Sequence Viewer 6.0.2 (CLC bio A/S, Aarhus, Denmark) software. However, the HBV genotype was also determined with the Basic Local Alignment Search Tool (BLASTN program 2.2.25, blast.ncbi.nlm.nih.gov) and by various genotyping tools; (I), The National Center for Biotechnology Information (NCBI, U.S National Library of Medicine, Bethesda - USA, www.ncbi.nih.gov), (II), The Genafor/Arevir - Geno2pheno Drug Resistance Tool (Center of Advanced European Studies and Research, Bonn - Germany, www.coreceptor.bioinf.mpi-inf.mpg.de), (III), and Stanford University HBV Drug Resistance Database (HBVseq: Sequence Analysis, hivdb.stanford.edu/HBV/HBVseq)

### 2.2. HBV Genotyping by Inno-Lipa

**Figure 1:**
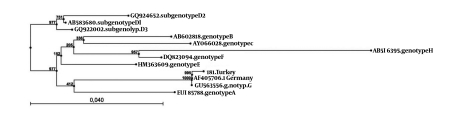
Phylogenetic Tree of Hepatitis B Virus Pol Gene (768 bp) Region. Neighbor-Joining Analysis Was Carried Out With Other Sequences From all HBV Genotypes From GenBank Using CLC Sequence Viewer 6.5.1 (CLC bio A/S, Aarhus, Denmark) Software. Bootstrap Support Value (1000 Replicates) are Shown at the Respective Branches. Strain Called 181 is Obtained From a Hemodialysis Patient (GenBank Accession Number.JN010438)

Extracted HBV DNA was amplified with biotinylated primers using AmpliTaq Gold DNA polymerase (Applied Biosystems, Inc., California, USA) and PCR buffer provided in the INNO-LiPA HBV genotyping kit (Innogenetics, Gent, Belgium) according to the manufacturer's instructions. The Lipa procedure was completed using the Auto-LiPA instrument (Innogenetics, Gent, Belgium). Phylogenetic analysis of the pol gene sequence showed that the isolate 181 clustered on a genotype G branch (Figure 1). The isolate 181 was also classified as genotype G according to subtyping tools (Table 1). The genotyping match and molecular features of the HBV isolate as indicated by the different genotyping tools are illustrated (Table 1). The biotinylated PCR product showed A + G mixed genotype infections with single reactive lines for genotype A and G on the INNO-LiPAHBV genotyping assay, respectively. After the pol gene sequence of the HBV isolate 181 was submitted, it was assigned a GenBank accession number: JN010438.

**Table 1:** Genotyping Match and Molecular Features of HBV Isolates on the Different Genotyping Tools

**Genotyping Tool**	**Genotype (Maximum Identity), %**	**Molecular Feature**
BLASTN	G, 94.0 a	Stop codon + frame shift b
NCBI	G, 98.0 c	ND d
HBV seq	G, 94.5	ND d
Geno2pheno	G, 96.6	Insertion
		In the surface protein, [ins.]148aT
		In the RT domain, [ins.]123aT
		Mutation e
		İn the surface protein, sM133T
		İn the RT domain, rtN238T

^a^ Maximum identity to strain with GenBank accession no: AF405706.1

^b^ The protein translation contains stop codons within the coding region and BLASTx similarity search results indicates these are frameshift. Analysis also on the Vector NTI v5.1 program has determined insertions (aT) on the base position numbers 148. and 123. in large S protein (a ORF, 492 bp) and RT domain (a ORF, 651 bp), respectively.

^c^ Maximum score was 284 to strain with GenBank accession no: AF405706.1

^d^ Not determined

^e^ sM133T mutation is a typical HBsAg escape mutation [17] . rtN238T is a compensatory mutation occure in adefovir therapy [18].

## 3. Discussion

This study reports the first recorded case of a Turkish patient being infected with the HBV genotype G. In 1990, an insertion of 36 nucleotides in the core region of the HBV characteristic of genotype G, was first identified in men who have had sex with other men [8]. Since then, this genotype has been identified as HBV genotype G in 13 chronic carriers from the Georgia area of the USA, and in France [1]. Genotype G HBV infection has frequently been reported with other HBV genotypes, such as genotype A (usually) or H (rare), but its clinical significance is not yet very clear due to limited reports [4][9][10]. Meanwhile, HBV genotype G coinfection with HIV has been described and reported from Brazil (2 out of 2 patients had HIV infection), Mexico (5 out of 5 patients had HIV infection), Germany (1 patient had HIV infection), France (3 out of 4 patients had HIV infection), Japan (2 out of 2 patients had HIV infection), and in the Cincinnati area of the USA (8 out of 33 patients had HIV infection) [2][3][11][12][13]. The present case study is an adult male, and his history of sexual relations with multiple partners may be identified as a risk factor for the transmission of HBV genotype G. The first HBV genotype G isolate in Mexico was obtained from a female sex worker [2], and since then it has been isolated from 4 men who had had sex with other men [10]. Various HBV genotype G strains were reported from the San Francisco area of the USA [9][14]. These strains could most probably have been acquired via sexual transmission. In a recently published report, detection with a specific method for HBV genotype G, on HBV-HIV coinfected patients, a 25% HBV genotype G prevalence was found, while it was below 1% in HBV monoinfections [15]. Nevertheless, there is insufficient information related to the sexual transmission of HBV genotype G and its association with HIV. The data previously obtained from Turkish patients indicated that the sequenced HBV did not show any genetic diversity. It has recently been reported that genotype D HBV is still predominant among Turkish patients who are chronically infected with HBV [5][6]. Those studies related HBV genotyping based on phylogenetic trees or subtyping tools and used population based sequencing. Meanwhile, the present case study showed A+G mixed genotype infection on the INNO-LiPA assay. This data demonstrates that sequence analysis is not the method of choice for determining the presence of mixed genotype infections. INNO-LiPA genotyping is based on reverse hybridization and is able to detect minor HBV subpopulations. Osiowy et al. reported that INNO-LiPA HBV genotyping assay was sensitive in detecting A+B, A+C and A+G mixed genotype infections compared with direct sequencing [16]. However, genotype G infection usually occurred as a coinfection with other HBV genotypes, therefore testing for the detection of genotype G should be included in the INNO-LiPA HBV genotyping assay. In summary, the genotype G HBV had never been reported before in Turkey, but our study suggests that the transmission of genotypes other than genotype D HBV is possible in Turkey. In the genotyping of HBV using phylogenetic analysis, various subtyping tools and the INNO-LiPA HBV genotyping assay may be more useful used in conjunction, for the identification of molecular features and to reveal coinfection with HBV genotype G, respectively. At the same time, circulation of the HBV genotype G strain is global, and genotype G is one of the least studied genotypes of HBV. Therefore in conclusion, it can be said that the identification and determination of the particular epidemiologic and molecular characteristics of the circulating genotype G strain of HBV is important to enable better understanding of the progression of chronic hepatitis infections around the world.
